# Effect of Tensile Stress Annealing on the Texture, Grain Size, and Magnetic Properties of Ultra-Thin Grain-Oriented Silicon Steel

**DOI:** 10.3390/ma18235416

**Published:** 2025-12-01

**Authors:** Chengzhou Niu, Ning Zhang, Yang Tu, Li Meng, Yong Yang

**Affiliations:** Metallurgical Technology Institute, Central Iron and Steel Research Institute Co., Ltd., Beijing 100081, China; niuchengzhoucall@163.com (C.N.); ty0171123688@163.com (Y.T.); li_meng@126.com (L.M.)

**Keywords:** ultra-thin grain-oriented silicon steel, annealing, tensile stress, texture, grain size, magnetic properties

## Abstract

This study systematically investigated the influence of annealing tension on the microstructure, texture, and magnetic properties of ultra-thin grain-oriented silicon steel, which is of great significance for achieving the preparation of high-quality ultra-thin grain-oriented silicon steel. The research indicates that tension primarily affects the magnetic properties by influencing the intensity of the η-fiber texture (<001>//RD) and the grain size during the annealing process, exhibiting a consistent trend across different annealing temperatures. That is, the proportion of η-oriented grains (or the intensity of the η-fiber texture) first decreased and then increased with increasing tension. Correspondingly, the magnetic induction (B_800_) decreased initially and then increased with the rise in annealing tension. Specifically, when annealed at 800 °C for 30 min, B_800_ decreased to 1.79 T under 24 MPa tension and then recovered to 1.86 T under 40 MPa tension. When annealed at 775 °C for 30 min, B_800_ decreased to 1.81 T under 24 MPa tension and subsequently recovered to 1.88 T under 40 MPa tension. In terms of grain size, the annealing tension promoted an increase in the average grain size. The synergistic effect of microstructure and texture led to a trend where the iron loss value (P_1.5/400_) of the ultra-thin strip under tension first increased and then decreased: when annealed at 800 °C for 30 min, the iron loss initially increased to 14.68 W/kg and then decreased with increasing tension; similarly, when annealed at 775 °C for 30 min, the iron loss first increased to 18.81 W/kg and then decreased with increasing tension. The evolution of the microstructure and texture is determined by the competition between the nucleation and growth of η-oriented grains and other grains during recrystallization: in the nucleation stage, the annealing tension reduced the strong advantage of η-oriented grains to some extent; however, it is speculated that η-oriented grains possess an advantage during the grain growth stage.

## 1. Introduction

Ultra-thin grain-oriented silicon steel offers advantages such as high magnetic induction and low medium-to-high frequency iron loss. It is primarily used in mid-to-high frequency applications ranging from 400 to 1000 Hz, including the electrical engineering sectors of ultra-high voltage power transmission and renewable energy grid integration equipment, as well as in defense, new energy vehicles, and high-efficiency motors. With the rapid development of ultra-high voltage direct current transmission and flexible alternating current transmission systems, there is an increasingly urgent demand for high-voltage, high-frequency, and high-capacity power equipment in the grid, leading to stricter requirements for the performance and quality of silicon steel materials used in this equipment [1,2,3,4,5,6,7].

For ultra-thin grain-oriented silicon steel with a thickness ≤ 0.10 mm, the current production method typically uses finished grain-oriented silicon steel sheets as the raw material (substrate), which are then processed through cold rolling and annealing treatments. Within the high-temperature environment of industrial continuous annealing furnaces, applying strip tension is necessary to ensure smooth processing. The setting and control of this tension are particularly critical: either insufficient or excessive tension can directly impact the product quality of subsequent annealing processes. Optimizing the design of the coiling tension control system in continuous annealing lines can significantly improve the precision and stability of tension control [8,9]. By applying precisely controlled tension, the stable suspended transportation of the strip inside the furnace can be effectively guaranteed, while simultaneously suppressing wrinkling and warping deformation of the strip at high temperatures, thereby improving strip flatness and geometric dimensional consistency [10]. However, the tension during the annealing process is often accompanied by changes in microstructure and texture, leading to a degradation of magnetic properties.

The influence of tension annealing on the microstructure, texture, and properties of metallic materials has been investigated to some extent. Cheng Guoping et al. [11] reported that for IF steel, with an increase in tension (0–750 N), the elongation and n-value decreased significantly. Mao Zhonghan et al. [12] found that during tension annealing and leveling of AgCu/QSn composite strips, applying a tension of 100–200 MPa within the temperature range of 200–350 °C for 1 min simultaneously improved the flatness and mechanical properties of the material. Murukawa Kei et al. [13] also discovered that an optimized tension annealing process could achieve a synergistic improvement in the flatness and mechanical properties of phosphor bronze sheets. Simulation results from Li Lin et al. [14] on 2205 duplex stainless steel indicated that under the conditions of 500 °C and 40 MPa tension annealing, the residual stress elimination efficiency was the highest, and the shape control effect was significant. Research by Ma Yonglin et al. [15] on cold-rolled SUS304 stainless steel strips demonstrated that the annealing tension significantly affected their mechanical properties and residual stress distribution. At an annealing temperature of 580 °C and an annealing tension of 6 MPa, the yield strength and tensile strength reached their maximum values, the internal residual stress distribution was the most uniform, and the strip shape was the best. This indicates that precise control of the tension annealing process window is crucial. Furthermore, it promoted grain growth while also promoting the {114}<481> orientation and suppressing the {111}<112> orientation, which is beneficial for texture optimization [16].

The effect of tensile stress annealing on the recrystallization behavior and texture of metallic materials has been investigated for various alloy systems. For Fe-3.0%Si non-oriented silicon steel, when the annealing tensile stress is less than 4 MPa, the average grain size increases with increasing annealing tensile stress. Concurrently, favorable texture components such as {001}<021> and {001}<100> are enhanced, while the {111}<112> and {111}<110> texture components are weakened, leading to reduced iron loss and increased magnetic induction in the silicon steel. However, when the annealing tensile stress is further increased to 6 MPa, the recrystallized grain size instead decreases [17,18]. Zhu Guangwei et al. [19] investigated the effect of tensile stress annealing on the recrystallization behavior and texture of Zr-4 alloy, finding that increasing the applied stress and annealing temperature promoted the recrystallization process, reduced the proportion of low-angle grain boundaries, and weakened the main texture components, thereby reducing the anisotropy of the material.

The aforementioned research has primarily focused on other alloy systems. For silicon steel, the existing studies concern non-oriented silicon steel with a thickness of 0.50 mm, and the range of tensile stress variation involved is relatively small. As the thickness of silicon steel sheets decreases, how annealing tensile stress affects the recrystallization process of ultra-thin grain-oriented silicon steel and consequently alters the evolution of its microstructure, texture, and magnetic properties remains unexplored. This study aims to investigate the effects of annealing tensile stress on the microstructure, texture evolution, and magnetic properties of ultra-thin grain-oriented silicon steel, which is of significant importance for achieving the preparation of high-quality ultra-thin grain-oriented silicon steel. Furthermore, this research can enrich and refine the understanding of the recrystallization behavior in silicon steel, including nucleation and grain growth behaviors, and can provide a reference for annealing control in other BCC-structured materials.

## 2. Materials and Methods

**Figure 1:**
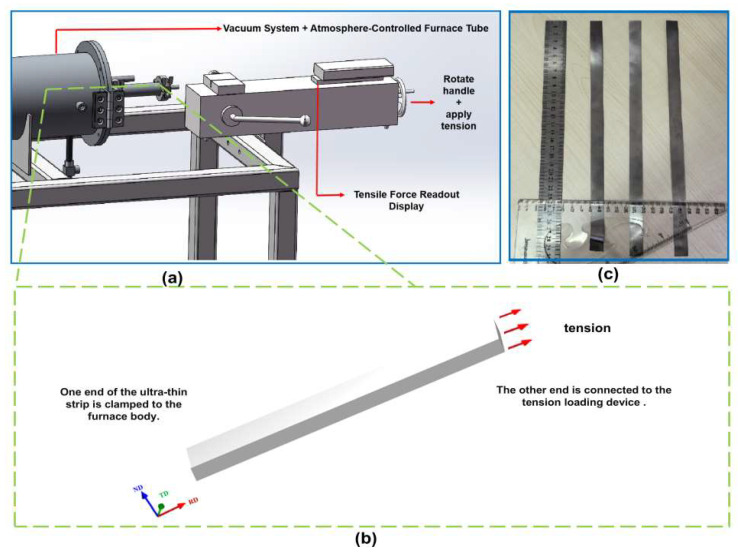
Atmosphere-protected tensile stress annealing process for the ultra-thin grain-oriented silicon steel strip. (**a**) Atmosphere-protected tensile stress annealing equipment; (**b**) schematic diagram of the tensile stress annealing process for the cold-rolled ultra-thin grain-oriented silicon steel strip; (**c**) the prepared 0.08 mm ultra-thin strip using the atmosphere-protected tensile stress annealing process.

The raw material used in this study was a finished grain-oriented silicon steel sheet with a thickness of approximately 0.35 mm. The chemical composition was determined using inductively coupled plasma atomic emission spectroscopy (ICP-AES) for Si, Mn, Al, and Sn contents, infrared absorption method for S and C contents, and thermal conductivity method for N content. The specific composition is listed in Table 1. Each element content was measured three times to ensure the repeatability and reproducibility of the results. After surface cleaning of the raw material, an ultra-thin grain-oriented silicon steel cold-rolled strip with a thickness of 0.08 mm was prepared via multi-roll cold rolling. Subsequently, the cold-rolled ultra-thin grain-oriented silicon steel samples underwent tension annealing treatment using an independently designed atmosphere-protected tensile stress annealing furnace. The tensile stress annealing equipment is shown in Figure 1a. The annealing furnace features a single-tube structure equipped with a precise temperature control system, vacuum system, atmosphere protection system, and tension loading device, enabling accurate control of the tensile stress during the high-temperature annealing process.

The annealing process was conducted under a pure hydrogen atmosphere inside the furnace. The annealing parameters were set as follows: annealing temperatures of 775 °C and 800 °C, a heating rate of 10 °C/min, and a soaking time of 30 min. A tension gradient was applied, set at 0, 8, 16, 24, 32, and 40 MPa. The annealing treatments were performed under a hydrogen protective atmosphere for 30 min to investigate the effect of the magnitude of the tensile stress on the recrystallized microstructure, texture, and magnetic properties. To clarify the role of tension during the recrystallization process, annealing treatments were also conducted on the cold-rolled ultra-thin strips at 750 °C for durations of 5 to 9 min, with the tensile stress levels set consistent with those used in the 775 °C and 800 °C annealing experiments.

The specific tensile stress annealing procedure is illustrated in Figure 1b. After the heating stage of the annealing furnace was completed, the ultra-thin grain-oriented silicon steel cold-rolled strip (with dimensions of 300 mm × 15 mm × 0.08 mm) was installed in a dedicated sintering boat. One end of the sample was fixed to the furnace body via a clamp, while the other end was connected to the tension loading device using a molybdenum wire. The length of the molybdenum wire was adjusted to ensure the sample remained straight within the furnace tube. Subsequently, a precise tensile stress was applied through the tension loading device. This applied stress was maintained until the conclusion of the annealing process. The tension loading device was then deactivated, allowing the sample to cool under a pure hydrogen atmosphere at a controlled cooling rate of 20 °C/min. Upon completion of the cooling stage, as shown in Figure 1c, the dimensions of the annealed sample were re-measured to monitor any elongation. It was confirmed that within the applied tension gradient range, the dimensional specifications of the ultra-thin grain-oriented silicon steel samples remained unchanged before and after the tensile stress annealing.

EBSD analysis was performed on samples at different annealing stages. The longitudinal sections of the samples were ground and mechanically polished, followed by etching with a 4% Nital solution. The rolled surfaces were initially ground with sandpaper and then electrophished using a solution of 15% perchloric acid in alcohol. Data acquisition and analysis, including orientation imaging maps (OIM), orientation distribution function (ODF) maps, and grain size information, were carried out using a Gemini SEM 300 field-emission scanning electron microscope (Zeiss, Oberkochen, Germany) equipped with a symmetry EBSD detector and HKL Channel 5 software. To ensure statistical reliability, three ultra-thin grain-oriented silicon steel strips (dimensions: 300 mm × 15 mm × 0.08 mm) from the same batch were prepared under each identical tensile stress annealing condition. From each strip, four EBSD test samples (dimensions: 10 mm × 8 mm) were sectioned at 75 mm intervals along the rolling direction. On each EBSD test sample, four scanning areas were selected at equal spacing. The specific EBSD acquisition parameters for each condition were as follows:

For samples annealed at 800 °C-30 min and 775 °C-30 min: Each scanning area measured 1137 μm × 852 μm with a step size of 3 μm, collecting data from approximately 2500 grains per area.

For samples annealed at 750 °C-5 min: Each scanning area measured 113.4 μm × 80.6 μm with a step size of 0.2 μm, collecting data from approximately 450 grains per area.

For samples annealed at 750 °C-7 min: Each scanning area measured 1136 μm × 165 μm with a step size of 1 μm, collecting data from approximately 1600 grains per area.

For samples annealed at 750 °C-9 min: Each scanning area measured 1137 μm × 175 μm with a step size of 1 μm, collecting data from approximately 850 grains per area.

Representative data from these analyses are presented in this paper.

A customized magnetic measurement system for ultra-thin silicon steel sheets was employed to determine the iron loss (P_1.5/400_) and magnetic induction (B_800_) of the ultra-thin grain-oriented silicon steel strips (dimensions: 300 mm × 15 mm × 0.08 mm) annealed at 775 °C for 30 min and 800 °C for 30 min under various tensile stresses. The measurement direction was parallel to the rolling direction. Considering the validity of experimental statistics, each sample was measured four times, and the average value was taken as the magnetic property for that specific sample.

## 3. Results

### 3.1. Effect of Annealing Tensile Stress on the Microstructure and Texture of Ultra-Thin Grain-Oriented Silicon Steel

**Figure 2:**
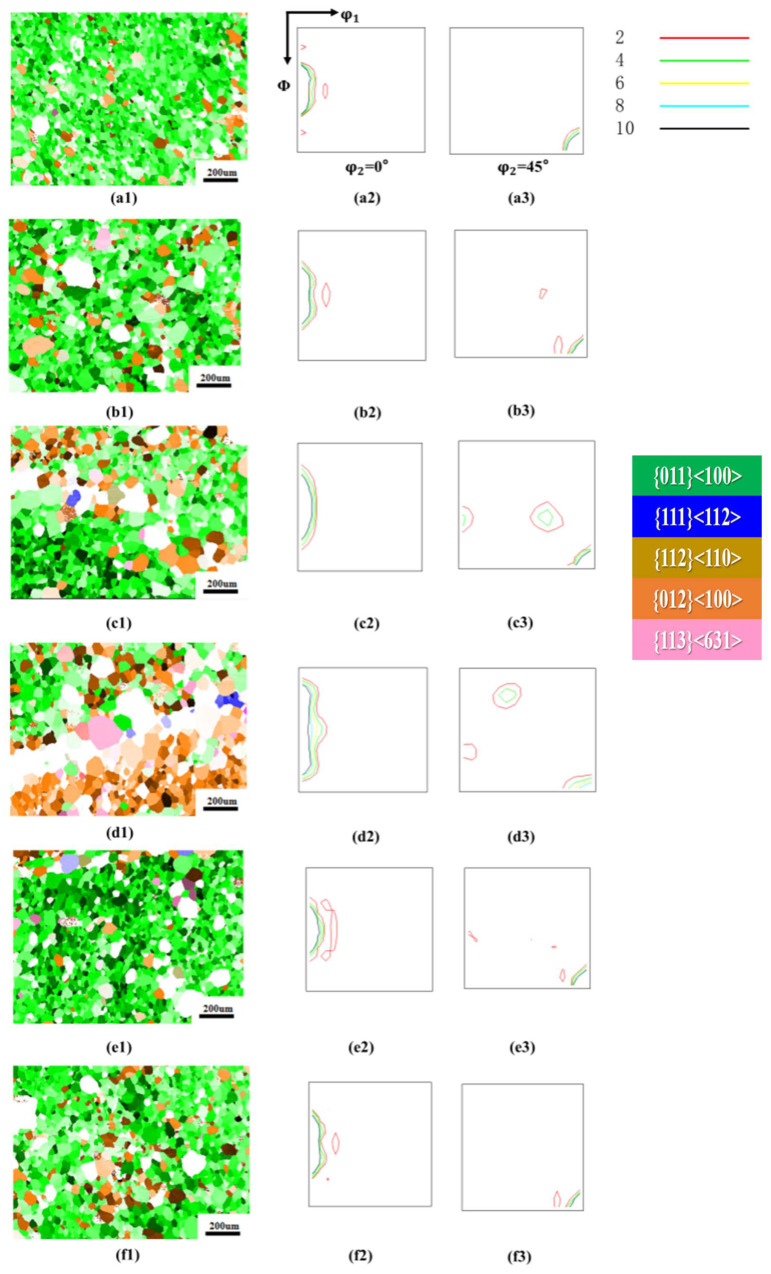
Microstructure and texture of the cold-rolled ultra-thin grain-oriented silicon steel strip after annealing at 800 °C for 30 min under different tensile stresses of 0–40 MPa. Tension 0 MPa: (**a1**) Typical orientation grain distribution map; (**a2**,**a3**) ODF maps (φ_2_ = 0° and φ_2_ = 45° sections); Tension 8 MPa: (**b1**) Typical orientation grain distribution map; (**b2**,**b3**) ODF maps (φ_2_ = 0° and φ_2_ = 45° sections); Tension 16 MPa: (**c1**) Typical orientation grain distribution map; (**c2**,**c3**) ODF maps (φ_2_ = 0° and φ_2_ = 45° sections); Tension 24 MPa: (**d1**) Typical orientation grain distribution map; (**d2**,**d3**) ODF maps (φ_2_ = 0° and φ_2_ = 45° sections); Tension 32 MPa: (**e1**) Typical orientation grain distribution map; (**e2**,**e3**) ODF maps (φ_2_ = 0° and φ_2_ = 45° sections); Tension 40 MPa: (**f1**) Typical orientation grain distribution map; (**f2**,**f3**) ODF maps (φ_2_ = 0° and φ_2_ = 45° sections).

Figure 2 shows the orientation imaging maps (OIM) and ODF sections (φ_2_ = 0° and 45°) of the 0.08 mm ultra-thin grain-oriented silicon steel cold-rolled strip after annealing at 800 °C for 30 min under different tensile stresses ranging from 0 to 40 MPa. It is evident that the annealing tensile stress exerts a non-monotonic influence on the evolution of the recrystallized microstructure and texture of the ultra-thin grain-oriented silicon steel. When no annealing tensile stress was applied (a1–a3), the recrystallized microstructure was primarily composed of η-oriented grains (including the Goss orientation {110}<001> and the {012}<100> orientation), which accounted for a high proportion. There were few non-η-oriented grains, the average grain size was relatively small, and the microstructure exhibited good uniformity. Upon applying an 8 MPa annealing tensile stress (b1–b3), the proportion of η-oriented grains slightly decreased, while non-η-oriented components such as {113}<631> increased to some extent. As the tensile stress increased to 16 MPa (c1–c3) and 24 MPa (d1–d3), the proportion of η-oriented grains continued to decrease, and the fraction of non-η-oriented grains rose significantly. The ODF maps indicated that the η-fiber texture became more diffuse. However, when the tensile stress was further increased to 32 MPa and 40 MPa (e1–f3), the proportion of η-oriented grains increased again.

**Figure 3:**
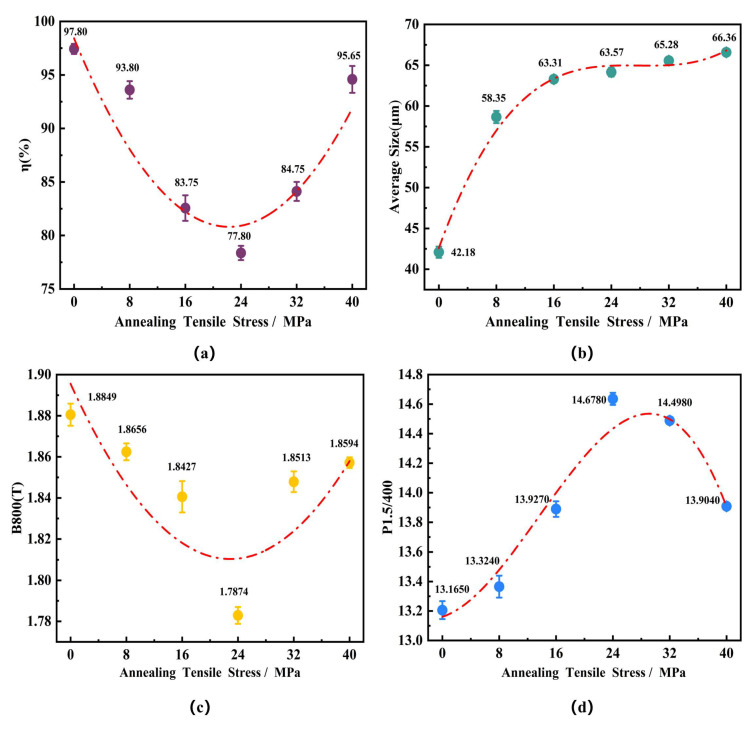
Microstructure, texture and magnetic properties of the ultra-thin grain-oriented silicon steel strip after annealing at 800 °C for 30 min under different tensile stresses of 0–40 MPa (**a**) Proportion of η-oriented grains; (**b**) Average grain size; (**c**) B_800_; (**d**) P_1.5/400_.

Related research has found that for ultra-thin grain-oriented silicon steel, the magnetic induction is primarily determined by the η-fiber texture [20]. Therefore, the proportion of the η-fiber texture was obtained through statistical analysis of three sets of data. Figure 3 quantitatively shows that the proportion of η-oriented grains (i.e., the intensity of the η-fiber texture) exhibited a trend of first decreasing and then increasing with increasing tensile stress. When the annealing tensile stress increased from 0 MPa to 24 MPa, the proportion of η-oriented grains decreased from 97.8% to 77.8%, a reduction of 20%. Once the tensile stress exceeded 24 MPa, the proportion of η-oriented grains began to increase, showing a significant rise particularly within the 32–40 MPa range, reaching 95.65% at 40 MPa, which was close to the level observed under tension-free annealing conditions. In terms of microstructure evolution, statistical analysis of the grain size using the linear intercept method revealed that the average grain size increased monotonically with increasing annealing tensile stress, from 42.18 μm at 0 MPa to 66.36 μm at 40 MPa. This indicates that the annealing tensile stress generally provided an additional driving force for the growth of recrystallized grains and promoted grain boundary migration.

**Figure 4:**
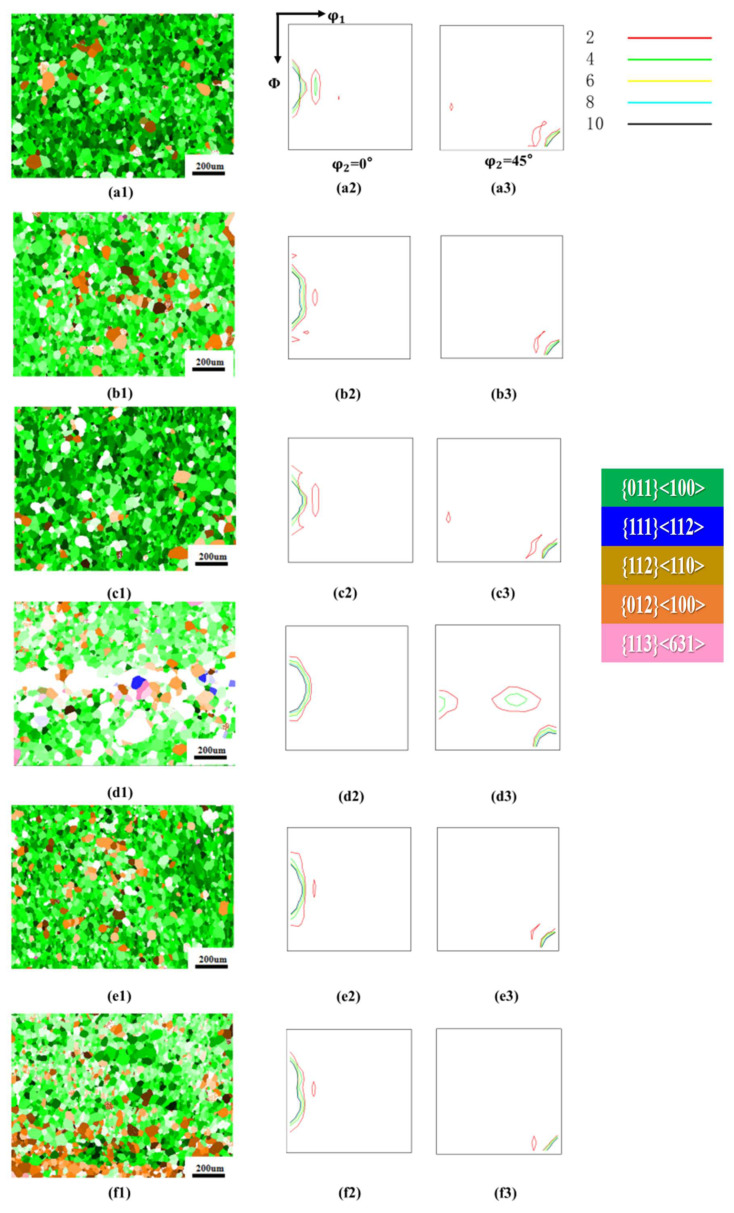
Microstructure and texture of the cold-rolled ultra-thin grain-oriented silicon steel strip after annealing at 775 °C for 30 min under different tensile stresses of 0–40 MPa. Tension 0 MPa: (**a1**) Typical orientation grain distribution map; (**a2**,**a3**) ODF maps (φ_2_ = 0° and φ_2_ = 45° sections); Tension 8 MPa: (**b1**) Typical orientation grain distribution map; (**b2**,**b3**) ODF maps (φ_2_ = 0° and φ_2_ = 45° sections); Tension 16 MPa: (**c1**) Typical orientation grain distribution map; (**c2**,**c3**) ODF maps (φ_2_ = 0° and φ_2_ = 45° sections); Tension 24 MPa: (**d1**) Typical orientation grain distribution map; (**d2**,**d3**) ODF maps (φ_2_ = 0° and φ_2_ = 45° sections); Tension 32 MPa: (**e1**) Typical orientation grain distribution map; (**e2**,**e3**) ODF maps (φ_2_ = 0° and φ_2_ = 45° sections); Tension 40 MPa: (**f1**) Typical orientation grain distribution map; (**f2**,**f3**) ODF maps (φ_2_ = 0° and φ_2_ = 45° sections).

Figure 4 shows the orientation imaging maps (OIM) and orientation distribution function (ODF) sections (φ_2_ = 0° and 45°) of the 0.08 mm ultra-thin grain-oriented silicon steel cold-rolled strip after annealing at 775 °C for 30 min under tensile stresses ranging from 0 to 40 MPa. Comparative analysis revealed that the influence of annealing tensile stress on the recrystallized microstructure and texture evolution of the ultra-thin grain-oriented silicon steel exhibited a highly consistent trend with that observed under the 800 °C annealing for 30 min condition. Furthermore, the proportion of η-oriented grainswas consistently higher than that under 800 °C annealing at identical stress levels. Under the condition of no applied tensile stress (a1–a3), the recrystallized microstructure was predominantly composed of η-oriented grains (including the Goss orientation {110}<001> and the {012}<100> orientation), with few non-η-oriented grains, a relatively small average grain size, and good microstructural uniformity. When an annealing tensile stress of 8 MPa (b1–b3) and 16 MPa (c1–c3) was applied, the proportion of η-oriented grainsslightly decreased, while non-η-orientedcomponents such as {113}<631> increased to some extent. As the tensile stress increased to 24 MPa (d1–d3), the proportion of η-oriented grainscontinued to decrease, and the fraction of non-η-oriented grainssignificantly increased; the ODF maps indicated that the η-fiber texturebecame more diffuse. However, when the tensile stress was further increased to 32 MPa and 40 MPa (e1–f3), the proportion of η-oriented grainsincreased again.

**Figure 5:**
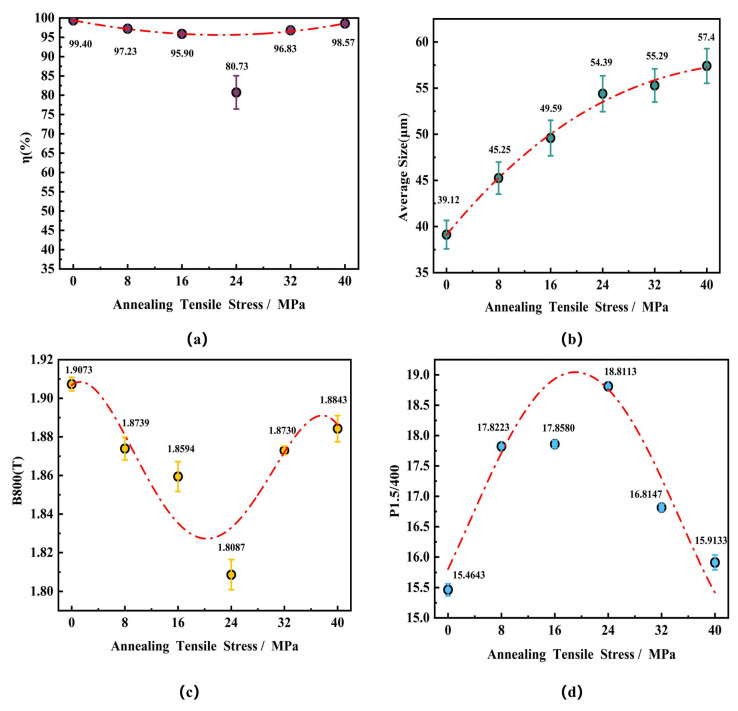
Microstructure, texture and magnetic properties of the ultra-thin grain-oriented silicon steel strip after annealing at 775 °C for 30 min under different tensile stresses of 0–40 MPa (**a**) Proportion of η-oriented grains; (**b**) Average grain size; (**c**) B_800_; (**d**) P_1.5/400_.

Figure 5 also quantitatively demonstrates a similar variation trend: the proportion of η-oriented grains (i.e., the intensity of the η-fiber texture) first decreased and then increased with increasing tensile stress, with 24 MPa serving as the inflection point. When the annealing tensile stress increased from 0 MPa to 16 MPa, the proportion of η-oriented grainsdecreased from 99.4% to 95.9%, a reduction of approximately 3.52%. As the stress continued to increase to 24 MPa, the proportion of η-oriented grainsdecreased further from 95.9% to 80.73%, representing a significant decline of about 15.82%. Once the tensile stress exceeded 24 MPa, the proportion of η-oriented grainsbegan to increase, showing a notable rise particularly within the 24–32 MPa range, where the increase amounted to approximately 20%. When the tensile stress was further increased to 40 MPa, the proportion of η-oriented grainsreached 98.57%. This trend is consistent with the behavior observed under 800 °C annealing with 40 MPa applied stress, where the proportion of η-oriented grainsalso returned to a level close to that of the tension-free annealed state. In terms of microstructural evolution, statistical analysis of the grain size using the linear intercept method confirmed that the average grain size increased monotonically with increasing annealing tensile stress, growing from 39.12 μm at 0 MPa to 57.4 μm at 40 MPa. This result further verifies that the annealing tensile stress generally provides an additional driving force for the growth of recrystallized grains and promotes grain boundary migration.

### 3.2. Effect of Annealing Tensile Stress on the Magnetic Properties of Ultra-Thin Grain-Oriented Silicon Steel

As shown in Figure 3, when annealed at 800 °C for 30 min, the applied tensile stress significantly influenced the magnetic properties of the ultra-thin grain-oriented silicon steel. The magnetic induction (B_800_) first decreased and then increased with increasing tension: it continuously decreased from 1.88 T under no applied stress to a minimum of 1.79 T at 24 MPa, representing a reduction of 4.8%. Once the tensile stress exceeded 24 MPa, B_800_ began to increase, reaching 1.86 T at 40 MPa; however, this value remained 1.06% lower than the measurement at 0 MPa. Conversely, the iron loss (P_1.5/400_) continuously increased with rising tensile stress from 13.17 W/kg under 0 MPa tension to a peak of 14.68 W/kg at 24 MPa, an increase of 11.47%. After the tensile stress exceeded 24 MPa, the iron loss slightly decreased to 13.90 W/kg at 40 MPa, yet it remained 5.5% higher than that in the stress-free state.

As shown in Figure 5, during annealing at 775 °C for 30 min under tensile stress, the magnetic induction (B_800_) of the ultra-thin grain-oriented silicon steel also exhibited a trend of first decreasing and then increasing with rising tension: it continuously decreased from 1.91 T under no applied stress to a minimum of 1.81 T at 24 MPa, representing a reduction of 5.23%. Once the tensile stress exceeded 24 MPa, B_800_ began to increase, reaching 1.88 T at 40 MPa; however, this value remained 1.57% lower than the B_800_ measurement obtained without annealing tension. The iron loss (P_1.5/400_) demonstrated a different response: it increased continuously with applied tension from 15.46 W/kg under 0 MPa stress to a peak of 18.81 W/kg at 24 MPa, representing a substantial increase of approximately 21.67%. Notably, within the low tensile stress range of 8 MPa to 16 MPa, the iron loss exhibited minimal variation with increasing annealing tension, showing only a marginal increase of 0.2%. After the tensile stress surpassed 24 MPa, the iron loss subsequently decreased to 15.91 W/kg at 40 MPa; nevertheless, this value remained 2.9% higher than that measured in the stress-free condition.

Therefore, it can be concluded that when the annealing tensile stress is maintained within an appropriate range, it facilitates the reduction of iron loss, enhances the magnetic induction, and thereby improves the final magnetic properties.

A strong correlation is observed between the magnetic induction and the recrystallization texture. The variation trend of B_800_ highly coincides with the intensity of the η-fiber texture. For samples annealed for 30 min, under tensile stress annealing at 800 °C, the minimum B_800_ value of 1.79 T at 24 MPa corresponds to the trough in the proportion of η-oriented grain sat 77.8%. Similarly, under tensile stress annealing at 775 °C, the minimum B_800_ value of 1.81 T at 24 MPa corresponds to the trough in the proportion of η-oriented grain sat 80.73%. This correspondence further confirms that the intensity of the favorable <001>//RD texture plays a dominant role in determining the magnetic induction. The variation in iron loss (P_1.5/400_), however, exhibits more complex characteristics. During tensile stress annealing at 800 °C, it reached a peak of 14.68 W/kg at 24 MPa and then decreased to 13.90 W/kg at 40 MPa. Correspondingly, during tensile stress annealing at 775 °C, it reached a peak of 18.81 W/kg at 24 MPa and subsequently decreased to 15.91 W/kg at 40 MPa. This change in iron loss reflects the synergistic effect of microstructure and texture. Within the grain size range of this study, the grain size increased from 42.18 μm under no tension to 66.36 μm at 40 MPa for the 800 °C annealing, and from 39.12 μm to 57.40 μm for the 775 °C annealing. This increase in grain size would benefit the reduction of iron loss by decreasing the hysteresis loss (P_h_). Simultaneously, a stronger favorable η-fiber texture also contributes to reducing the iron loss. Overall, the maximum iron loss occurred when the proportion of η-oriented grains was at its minimum, and it gradually decreased with the recovery of the η-fiber texture intensity and the increase in grain size.

Under the experimental conditions of this study, the influence of annealing tension on the magnetic properties of the ultra-thin grain-oriented silicon steel strip was related to the annealing temperature. When annealed at 800 °C, a relatively small annealing tension (8 MPa) was beneficial for optimizing both magnetic induction and iron loss, with values only slightly higher than those obtained without applied tension. In contrast, during annealing at 775 °C, larger annealing tensions, such as 32 MPa and 40 MPa, were conducive to reducing iron loss while simultaneously enhancing magnetic induction. Throughout this process, the stress applied during tensile stress annealing is likely the reason why the iron loss of tension-annealed samples is generally higher than that of samples annealed without tension.

### 3.3. Effect of Annealing Tensile Stress on the Recrystallization Nucleation Behavior of Ultra-Thin Grain-Oriented Silicon Steel

**Figure 6:**
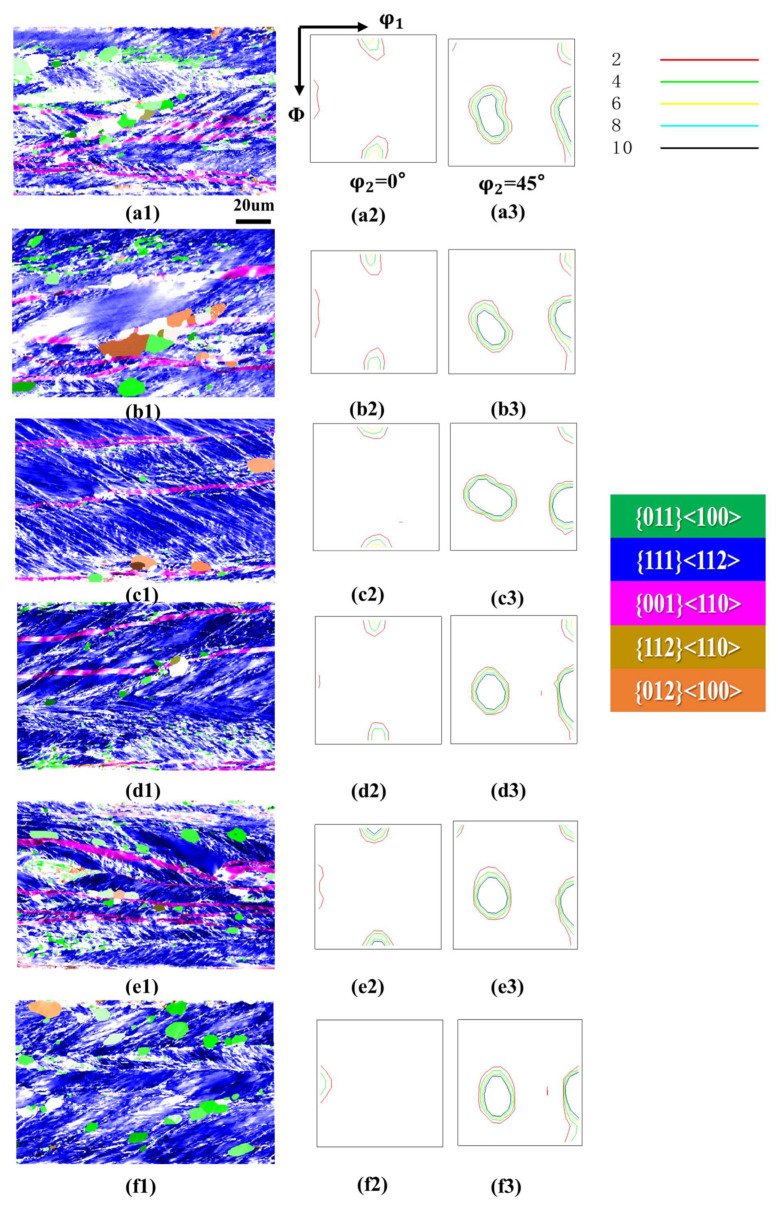
Microstructure and texture of the cold-rolled ultra-thin grain-oriented silicon steel strip after annealing at 750 °C for 5 min under different tensile stresses of 0–40 MPa. Tension 0 MPa: (**a1**) Typical orientation grain distribution map; (**a2**,**a3**) ODF maps (φ_2_ = 0° and φ_2_ = 45° sections); Tension 8 MPa: (**b1**) Typical orientation grain distribution map; (**b2**,**b3**) ODF maps (φ_2_ = 0° and φ_2_ = 45° sections); Tension 16 MPa: (**c1**) Typical orientation grain distribution map; (**c2**,**c3**) ODF maps (φ_2_ = 0° and φ_2_ = 45° sections); Tension 24 MPa: (**d1**) Typical orientation grain distribution map; (**d2**,**d3**) ODF maps (φ_2_ = 0° and φ_2_ = 45° sections); Tension 32 MPa: (**e1**) Typical orientation grain distribution map; (**e2**,**e3**) ODF maps (φ_2_ = 0° and φ_2_ = 45° sections); Tension 40 MPa: (**f1**) Typical orientation grain distribution map; (**f2**,**f3**) ODF maps (φ_2_ = 0° and φ_2_ = 45° sections).

**Figure 7:**
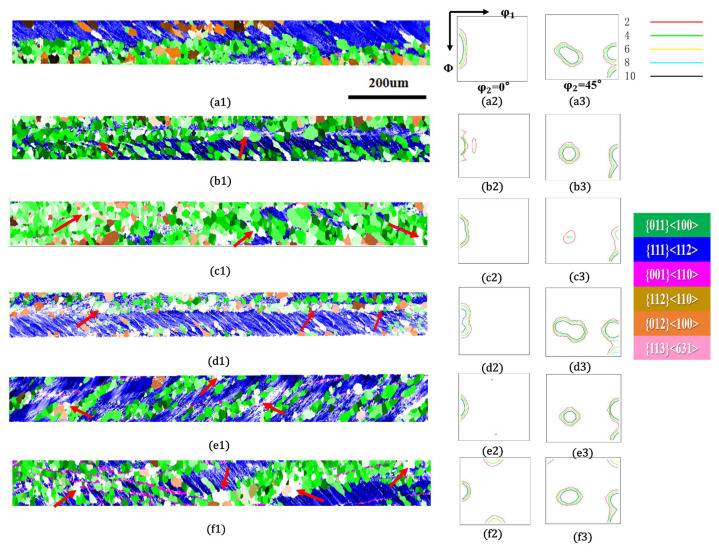
Microstructure and texture of the cold-rolled ultra-thin grain-oriented silicon steel strip after annealing at 750 °C for 7 min under different tensile stresses of 0–40 MPa. Tension 0 MPa: (**a1**) Typical orientation grain distribution map; (**a2**,**a3**) ODF maps (φ_2_ = 0° and φ_2_ = 45° sections); Tension 8 MPa: (**b1**) Typical orientation grain distribution map; (**b2**,**b3**) ODF maps (φ_2_ = 0° and φ_2_ = 45° sections); Tension 16 MPa: (**c1**) Typical orientation grain distribution map; (**c2**,**c3**) ODF maps (φ_2_ = 0° and φ_2_ = 45° sections); Tension 24 MPa: (**d1**) Typical orientation grain distribution map; (**d2**,**d3**) ODF maps (φ_2_ = 0° and φ_2_ = 45° sections); Tension 32 MPa: (**e1**) Typical orientation grain distribution map; (**e2**,**e3**) ODF maps (φ_2_ = 0° and φ_2_ = 45° sections); Tension 40 MPa: (**f1**) Typical orientation grain distribution map; (**f2**,**f3**) ODF maps (φ_2_ = 0° and φ_2_ = 45° sections).

**Figure 8:**
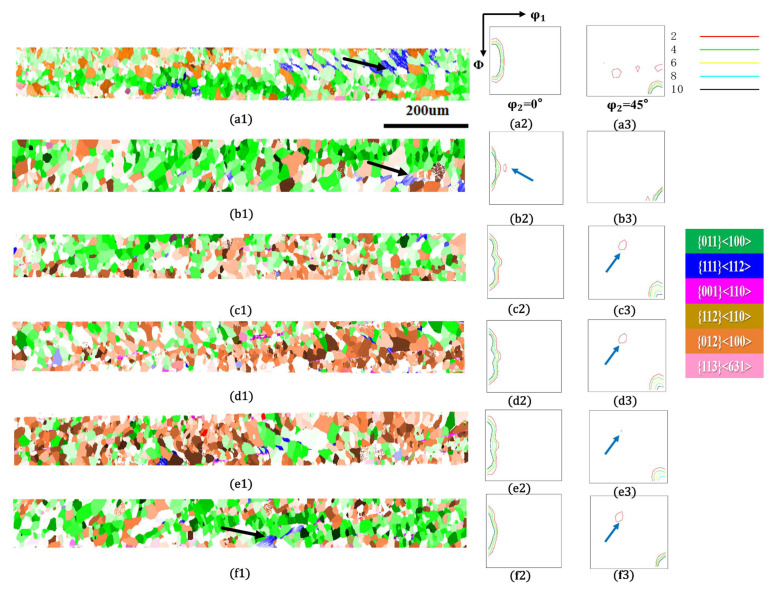
Microstructure and texture of the cold-rolled ultra-thin grain-oriented silicon steel strip after annealing at 750 °C for 9 min under different tensile stresses of 0–40 MPa. Tension 0 MPa: (**a1**) Typical orientation grain distribution map; (**a2**,**a3**) ODF maps (φ_2_ = 0° and φ_2_ = 45° sections); Tension 8 MPa: (**b1**) Typical orientation grain distribution map; (**b2**,**b3**) ODF maps (φ_2_ = 0° and φ_2_ = 45° sections); Tension 16 MPa: (**c1**) Typical orientation grain distribution map; (**c2**,**c3**) ODF maps (φ_2_ = 0° and φ_2_ = 45° sections); Tension 24 MPa: (**d1**) Typical orientation grain distribution map; (**d2**,**d3**) ODF maps (φ_2_ = 0° and φ_2_ = 45° sections); Tension 32 MPa: (**e1**) Typical orientation grain distribution map; (**e2**,**e3**) ODF maps (φ_2_ = 0° and φ_2_ = 45° sections); Tension 40 MPa: (**f1**) Typical orientation grain distribution map; (**f2**,**f3**) ODF maps (φ_2_ = 0° and φ_2_ = 45° sections).

The characteristics of the recrystallized microstructure and texture in ultra-thin grain-oriented silicon steel strips are determined by the recrystallization nucleation and growth behaviors. Figure 6, Figure 7 and Figure 8 show the evolution process of the 0.08 mm grain-oriented silicon steel cold-rolled strip after annealing at 750 °C for 5–9 min. To better evaluate the shear band nucleation occurring in this material, EBSD characterization was performed on the longitudinal section of the cold-rolled and annealed strip. As shown in the orientation imaging map in Figure 6, the sample annealed for 5 min was in the initial stage of recrystallization nucleation. At this stage, nucleation predominantly occurred via shear bands, with preferentially nucleated orientations being η-orientations such as Goss and {012}<100>, while the deformed matrix consistently exhibited a {111}<112> orientation [21]. The η and {111}<112> texture components can also be observed in the ODF sections at φ_2_ = 0° and 45°.

When the annealing holding time was extended to 7 min, as shown in Figure 7, nucleation occurring on shear bands, primarily of the η-fiber type, still dominated in all samples. The recrystallization nucleation behavior of the ultra-thin grain-oriented silicon steel is first directly affected by the orientation of the deformed structure. The orientation of the deformed structure in Figure 7(d1–d3) deviates considerably from the standard {111}<112> orientation; consequently, nucleation in this region occurs later than in other areas. Furthermore, among the nucleated grains in this region, Goss-oriented grains are relatively few, with a preference instead for the {210}<001> orientation [21,22]. It is noteworthy that compared with Figure 7(a1–a3), an increase in non-η-oriented nucleated grains was observed in Figure 7(b1–f3) under applied tensile stress (indicated by red arrows). This phenomenon is particularly significant in Figure 7(c1–d3,f1–f3). As mentioned previously, the orientation of nucleated grains is largely influenced by the orientation of the deformed matrix. However, under the same deformed matrix, the proportion of non-η-oriented nucleated grains in Figure 7(c1–c3,f1–f3) is significantly higher than that in the sample without applied tension (Figure 7(a1–a3)). It is speculated that the presence of tensile stress during the annealing process promotes the nucleation of non-η-oriented grains.

After the annealing holding time was further extended to 9 min, as shown in Figure 8, nucleation was nearly complete in each region, with only a small amount of un-nucleated γ-fiber texture remaining (as indicated by black arrows). Among them, the sample without applied annealing tensile stress (Figure 8(a1–a3)) had the most remaining deformed structure, thus presenting a certain γ-fiber texture in the ODF map. Comparing the recrystallized microstructure and texture of each sample, it can be seen that all samples are dominated by the η-fiber recrystallization texture, but the presence of non-η-fiber texture can be observed in all tension-annealed samples, such as the component near {h,1,1}<1/h,1,2> indicated by blue arrows in the ODF diagram. This component is generally stated to originate from α-fiber deformed grains, which is related to the heterogeneous strain accumulation at grain boundaries [23], and it has been observed to be promoted in the primary recrystallization structure of tension-annealed grain-oriented silicon steel [16]. This is consistent with the phenomenon observed in Figure 7, where some nucleations that originally did not have an advantage appeared under tension and eventually formed texture components.

## 4. Discussion

**Figure 9:**
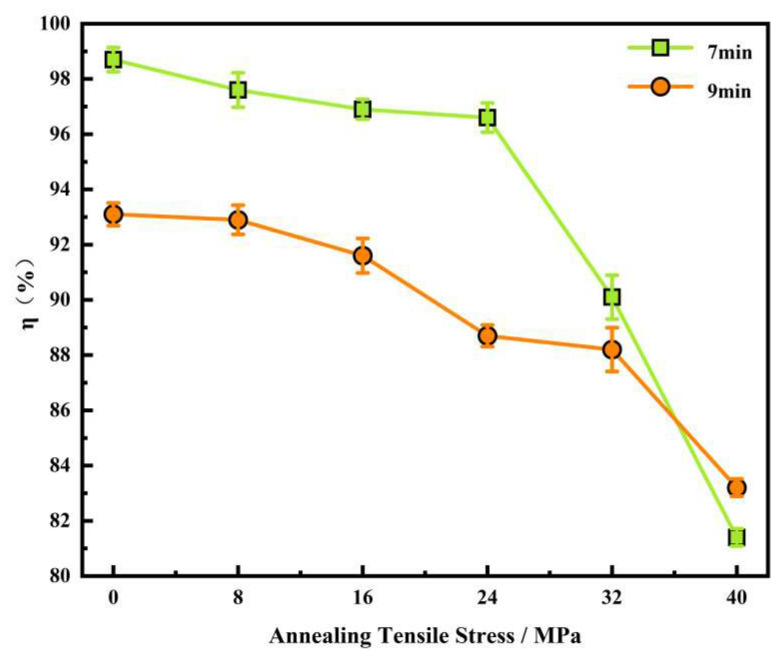
Proportion of η-oriented grains within nucleated grains in the cold-rolled ultra-thin grain-oriented silicon steel strip after annealing at 750 °C for 7 min and 9 min under different tensile stresses of 0–40 MPa.

Figure 9 shows the statistical results of the proportion of η-oriented grains within the nucleated grains during the annealing process of the 0.08 mm ultra-thin grain-oriented silicon steel cold-rolled strip at 750 °C for 7 min and 9 min, respectively, with simultaneous application of 0~40 MPa tensile stress. For the nucleation of ultra-thin grain-oriented silicon steel, η-oriented grains always maintain a strong advantage, with this study showing an even stronger advantage at the relatively lower temperature of 775 °C. However, the presence of tensile stress may increase the nucleation capability of grains with various orientations, such as the {113} and other non-typical oriented grains observed in Figure 7 and Figure 8, consequently reducing the relative advantage of η-oriented grains. As the annealing tensile stress gradually increased from 0 MPa to 40 MPa, the proportion of η-oriented grains among all newly nucleated grains showed a progressive downward trend. When no tensile stress was applied, the proportion of η-oriented grains within the nucleated grains was as high as 98.7% (7 min) and 93.1% (9 min). In the tensile stress range of 0~24 MPa, the proportion of η-oriented grains slightly decreased but remained at a relatively high level, with values of 96.6% (7 min) and 88.7% (9 min). However, when the tensile stress increased to 32 MPa and 40 MPa, the proportion of η-oriented grains within the nucleated grains significantly decreased to approximately 80%.

Comparative analysis of the experimental results from Figure 9 (750 °C/7 min, 9 min) with Figure 3 (800 °C/30 min) and Figure 5 (775 °C/30 min) reveals that although the proportion of η-oriented grains in the nucleated grains under high tension conditions (32–40 MPa) is relatively low, after complete recrystallization annealing, the proportion of η-oriented grains in the final microstructure still recovers to a high level of 95.65% (see Figure 3) and 98.57% (see Figure 5). Compared to the process without applied tension, the difference during tension annealing lies in the fact that tension can increase grain boundary mobility, thereby promoting an overall increase in grain size [24,25,26,27,28], as shown in Figure 3 and Figure 5. Furthermore, the presence of tension has an orientation-dependent influence on the growth of grains with different orientations [29,30]. For this study, since the η-oriented grains have <001>//RD and are thus parallel to the tensile stress direction, in BCC-structured silicon steel, the <001> crystallographic direction has a lower elastic modulus [31], and energy tends to release easily, leading to a higher developing ability during grain growth.

**Figure 10:**
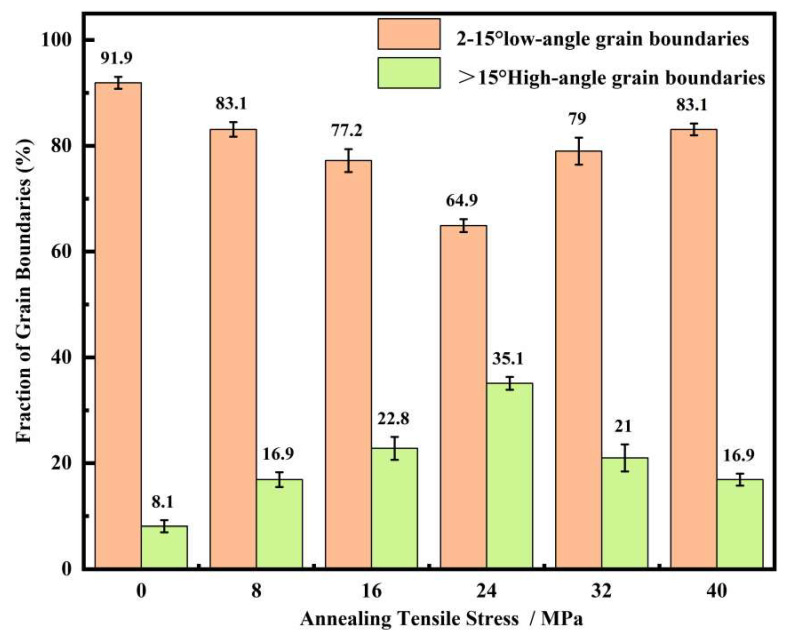
Proportion of low-angle and high-angle grain boundaries in the cold-rolled ultra-thin grain-oriented silicon steel strip after annealing at 850 °C for 30 min under different tensile stresses of 0–40 MPa.

Based on the aforementioned analysis, the application of tension is beneficial for increasing the grain size of all grains, especially the η-oriented grains. However, as seen from Figure 3 and Figure 5, the rate of increase in the average grain size of the annealed samples slowed down with increasing tension. The grain growth process in the ultra-thin strip of grain-oriented silicon steel is determined by the combined effects of the growth and clustering of η-oriented grains and the growth of non-η-oriented grains [32]. Among these, the dominant η-oriented grains have limited growth ability due to the texture inhibition effect, and they gradually cluster into clusters. Figure 10 shows the distribution of grain boundaries in the sample after annealing at 800 °C for 30 min. It can be observed that in the absence of applied tension, the annealed sample exhibits the highest proportion of low-angle grain boundaries. With the application of tension, the proportion of low-angle grain boundaries first decreases and then increases. This trend is consistent with the variation in the proportion of η-oriented grains within the samples, as a large number of low-angle grain boundaries exist between the η-oriented grains. The significant presence of these low-angle grain boundaries indicates that grain growth is inhibited to some extent, meaning the promoting effect of tension on grain growth is somewhat weakened. In summary, the annealing tension simultaneously influences both the nucleation and growth processes of recrystallization. The competitive relationship between grains of different orientations during nucleation and growth determines the evolution of the microstructure and texture. During the nucleation stage, the presence of tension somewhat weakens the strong nucleation advantage of the η-oriented grains. In the subsequent growth stage, the η-oriented grains possess a stronger growth driving force, gradually restoring their textural advantage. Additionally, the effect of texture inhibition cannot be neglected in the grain growth process of ultra-thin grain-oriented silicon steel.

## 5. Conclusions

This study systematically investigated the effects of annealing tension on the evolution of recrystallized microstructure, texture, and magnetic properties of 0.08 mm ultra-thin grain-oriented silicon steel. The main conclusions are as follows:

(1) Annealing tension influences the magnetic properties by affecting the evolution of the recrystallized microstructure and texture. During annealing at different temperatures, the proportion of η-oriented grains (<001>//RD) exhibited a similar trend, first decreasing and then increasing with increasing tension, reaching its lowest value under 24 MPa of tension: namely, 77.8% at 800 °C and 80.73% at 775 °C. Correspondingly, the magnetic induction showed a trend of first decreasing and then increasing with the increase in annealing tension. Regarding grain size, the annealing tension promoted an increase in the average grain size. However, the initial weakening and subsequent strengthening of the η-fiber texture with increasing tension caused the core loss of the ultra-thin strip to first increase and then decrease.
(2) The influence of annealing tension on the microstructure and texture originates from its effect on the differential behavior between η-oriented grains and other grains during recrystallization. During the nucleation stage, the strong nucleation advantage of η-oriented grains is weakened to some extent by the presence of annealing tension, leading to a decrease in the proportion of newly nucleated grains with the η-orientation as the tension increases. However, the η-fiber texture in the ultra-thin strips annealed at 800 °C and 775 °C for 30 min was enhanced under high tension, which suggests a growth advantage for η-fiber-recrystallized grains under annealing tension. The grain growth process in ultra-thin grain-oriented silicon steel is also constrained by texture inhibition effects. The presence of a large number of low-angle grain boundaries between η-oriented grains suppresses their growth capability to some extent.
(3) Under the experimental conditions of this study, a rationale for selecting the annealing tension for ultra-thin grain-oriented silicon steel is proposed. For annealing at 800 °C, a relatively small annealing tension (8 MPa) is beneficial for optimizing both the magnetic induction and core loss. In contrast, for annealing at 775 °C, a larger annealing tension is advantageous for reducing the core loss and improving the magnetic induction.

## Figures and Tables

**Table 1:** Chemical Composition of Finished Grain-Oriented Silicon Steel Sheet (wt/%).

Chemical Elements	C	Si	Mn	S	Al	N	Sn
Mass Fraction	0.0058	3	0.0088	0.0003	0.005	0.001	0.1

## Data Availability

The data presented in this study are available on request from the corresponding author due to ongoing research and further analysis.
